# Long Non-coding RNA Gas5 Is Associated With Preeclampsia and Regulates Biological Behaviors of Trophoblast via MicroRNA-21

**DOI:** 10.3389/fgene.2020.00188

**Published:** 2020-03-03

**Authors:** Dongying Zheng, Yue Hou, Yuanyuan Li, Yue Bian, Muhanmmad Khan, Fan Li, Ling Huang, Chong Qiao

**Affiliations:** ^1^Department of Obstetrics and Gynecology, Shengjing Hospital, China Medical University, Shenyang, China; ^2^Department of Obstetrics and Gynecology, Second Affiliated Hospital of Dalian Medical University, Dalian, China; ^3^Key Laboratory of Maternal-Fetal Medicine of Liaoning Province, Shenyang, China; ^4^Key Laboratory of Obstetrics and Gynecology of Higher Education of Liaoning Province, Shenyang, China; ^5^Research Center of China Medical University Birth Cohort, Shenyang, China; ^6^Department of Zoology, University of the Punjab, Lahore, Pakistan

**Keywords:** long non-coding RNA, Gas5, preeclampsia, placenta, trophoblast, miR-21, early-onset

## Abstract

Preeclampsia is a lethal pregnancy specific hypertensive disorder involving multisystem. Despite extensive studies to investigate the causes of preeclampsia, the pathogenesis still remains largely unknown. Long non-coding RNAs (lncRNAs) are a diverse class of non-translated RNAs which play a crucial part in various biological phenomena. Although lncRNA Growth Arrest-Specific 5 (GAS5) aberrantly expressed in multiple cancer tissues and is implicated in multiple biological processes of tumor cells, little is known about its role in preeclampsia. In this study, 40 patients with preeclampsia and 32 gestational age matched normotension pregnant women were recruited. Using quantitative real-time polymerase chain reaction (qRT-PCR), we found higher expression of GAS5 in placenta of preclamsia affected women. The level of GAS5 existed strongly in correlation with Thrombin Time indicating coagulation function and other clinical parameters by Pearson correlation analysis. Then we constructed the GAS5 lentivirus expression vectors and transfected into human trophoblast cell lines HTR-8/SVneo and JEG-3. Using *in vitro* cell culture studies, we found an inhibited effect of GAS5 on proliferative ability, migratory ability and invasive ability however; no effect on apoptosis was detected. Further mechanistic analysis found that GAS5 modulated microRNA-21 (miR-21) in an opposite variation tendency by qRT-PCR and rescue experiment. In addition, inhibition of GAS5 promoted the activation of PI3K/AKT signaling pathway and its downstream proteins covering MMP-9 and TP53 as evident from our qRT-PCR and western blot analyses. Thus, we suggested that GAS5 might involve in pregnancy with preeclampsia by influencing the biological functions of trophoblast cells through the regulation of miR-21 and activation of PI3K/AKT signaling pathway and its downstream targets, which may contribute to reveal the nature of preeclampsia.

## Introduction

Preeclampsia (PE) is a pregnancy specific multisystem hypertensive disorder that affects 3–5% of pregnancies, a major contributor to maternal, neonatal morbidity and mortality (Mol et al., 2016). The incidence of preeclampsia reported in China in recent years is 2.4–4.2% (Xiao et al., 2014) which still fluctuates greatly for the lack of prevention and treatment in economically underdeveloped regions. Despite advances in technology, termination of pregnancy is still the only effective treatment which induces iatrogenic preterm birth and adverse maternal and fetal outcomes. Furthermore, preeclampsia also confers an increased long-term risk of cardiovascular disease in both the mothers and offspring (Bokslag et al., 2016; Stanhewicz, 2018).

The pathogenesis of preeclampsia is still poorly understood, spans the fields of genetics, immunology and endocrinology. The two-stage model of preeclampsia is the most widely accepted central hypothesis. In normal pregnancy, cytotrophoblasts translocate from the chorionic villi into the uterus endometrium, achieving the inner third of the myometrium. Within the myometrium, cytotrophoblasts invade deeply the spiral arteries to replace the lining of maternal endothelia and interpose themselves amongst the vascular smooth muscle cells. As a result, the spiral arteries attain adequately perfusion for the placenta. On the contrary, the invasion of cytotrophoblasts is frequently shallow and incomplete in preeclampsia. The insufficient trophoblast invasion in first stage causes placental ischemia and hypoxia, the released damaging factors effecting on systemic vascular endothelial cell induces the clinical symptoms in the second stage (Fisher, 2015). To date, the main investigative focus is the initial stage of the disease-trophoblast invasion disorder.

Preeclampsia is distinguished into different subtype for better diagnosis and treatment. Early-onset (gestational weeks less than 34 weeks) versus late-onset (gestational weeks are equal to or more than 34 weeks) is one of the categories based on the time of disease onset. Most researches have indicated early-onset preeclampsia linked to poor placentation, whereas late-onset is suggested to result from preexisting maternal factors (e.g., obesity, diabetes mellitus, chronic hypertension, and some autoimmune diseases). Despite the identical diagnostic criteria and overlapped presenting features, they are associated with different clinical features and outcomes of mothers and fetuses. Early-onset preeclampsia is associated with significantly greater risk of maternal and fetal complications which often deteriorates rapidly (Phipps et al., 2016; Wojtowicz et al., 2019).

Human genome can be transcribed into protein-coding genes and non-coding RNAs with advances in high-throughput genomic technologies. Given the complex pathways implicated in preeclampsia, numbers of studies have been conducted to examine expression differences of genes in preeclampsia placentas. These studies have largely been successful in finding significant differential expression of protein-coding genes in preeclampsia individuals. However, only 1–2% of the genome is transcribed into proteins contrasted to 75% of the genome forms non-coding RNA transcripts during biological activities (Wilusz, 2016). Even though studies to explore the placental expressive discrepancy of non-coding RNAs between preeclampsia patients and normal pregnant women increased year by year, considering its enormous amount, there is clearly much work to be done, especially in mechanistic analysis.

These non-coding RNAs are comprised of long non-coding RNAs (lncRNA) and small non-coding RNAs [including microRNA (miRNA)] according to their nucleotides length. LncRNAs are more than 200 nucleotides in length. Since the next generation sequencing had been introduced, there is a steady expansion in the quantity of lncRNAs identified so far. LncRNAs have various molecular functions. They can perform as antisense decoys, signals and scaffolds, likewise, engaging in interferences of transcription. Although the number of lncRNAs identified increases continuously, the biological significances and functions of the majorities remain unknown. They promisingly and strongly influence the development and health, associated with a variety of human complicated diseases (McAninch et al., 2017).

The function of lncRNAs in the development of placenta is still acquainted scarcely, mainly speculated from studies on placental pathologies. Transcriptomic analyses of total RNAs from placenta and decidua allowed the identification of differential lncRNAs expression between preeclampsia and control pregnant women, often accompanying with a difference between Early and late-onset. The majorities of these lncRNAs had been identified in the research of cancers previously, frequently associated with cellular invasion, migration, and proliferation. Given the similar features between trophoblasts and cancer cells during placentation such as rapid proliferation, migration and invasion of the maternal tissues, extensive *in vitro* research studies have been conducted to elucidate the roles of these lncRNAs in trophoblast physiology. A variety of lncRNAs including H19, MALAT-1, MEG3, RNA-ATB. PVT1, TUG1, and DIAPH2-AS1 involved in preeclampsia and were discussed in detail and in-depth (Apicella et al., 2019).

lncRNA GAS5 is a transcript of the growth arrest-specific 5 (*gas5*) gene. “*Gas5* is localized at 1q25.1 and comprises 12 exons, which contain only a short open reading frame and are not thought to encode a functional protein. It was first isolated in 1988 in a search for novel tumorous suppressors by subtractive cDNA cloning of genes which are preferentially expressed in growth-arrested cells. The exons of *gas5* are spliced to yield two possible mature lncRNAs, termed GAS5a and GAS5b, due to the presence of alternative 5′-splice donor sites in exon 7” (Pickard and Williams, 2015). Mature lncRNA appears to be the predominant transcript in most cell lines, and particularly the GAS5b variant. *Gas5* additionally encodes within its introns 10 box C/D snoRNAs, which participate in the 2′-*O*-methylation of ribosomal RNA (Pickard and Williams, 2015). Recent work has shown that lncRNA GAS5 is dysregulated in multiple human cancers and confirms a tumor suppressor role (Liu et al., 2013; Zhang et al., 2013; Wang et al., 2018). GAS5 can act as the molecular spongy binding numerous microRNAs mutually but the observations in detail are still remained largely unclear. Negative regulation between GAS5 and microRNA-21 (miR-21) has been certificated in various tumors and other diseases for the GAS5 Exon 4 contains miR-21 binding sequence (Zhang et al., 2013; Song et al., 2014; Hu et al., 2016; Cao et al., 2017; Tao et al., 2017; Suo et al., 2018; Yao et al., 2019).

Despite the achievement in field of tumor research, there are few studies about GAS5 on pregnancy related diseases. Using RNA fluorescent *in situ* hybridization methods, GAS5 was firstly identified in syncytiotrophoblasts of human third trimester placentas. For the purpose of revealing interaction between GAS5 and prenatal maternal stress, BeWo and JEG-3 cytotrophoblast cell lines were treated with gradient concentration of cortisol, and expression of GAS5 was significantly upregulated (Mparmpakas et al., 2014). Until now, role of GAS5 in preeclampsia has not been reported. Previous work of our research group showed that in GEO Profiles, high throughput sequencing affirmed the high level of GAS5 expressed in human placental tissue and the different expression between preeclampsia and normal pregnancy. Therefore, this research firstly attempted to verify the GAS5 involvement of preeclampsia and the correlation between GAS5 and clinical parameters. Subsequently, we constructed GAS5 lentivirus overexpression and shRNA knockdown vectors to detect the impact of GAS5 on the biological behavior of trophoblast cells. Finally, GAS5 was proved by targeting miR-21 to affect PI3K/AKT (phosphatidylinositol 3-kinase/serine-threonine kinase) signaling pathway and the downstream proteins. Thus the relevant mechanism of GAS5 involved the preeclampsia pathogenesis was preliminarily explored.

## Materials and Methods

The study was conducted at Shengjing Hospital of China Medical University. The placenta tissue collection and preparation is under the local Ethics policy and the institutional review board approval of Medical Faculty Committee. All participants included in the study provided informed consents.

### Study Population

The subject of the research only includes singleton pregnant women admitted at Obstetrics Department in Shengjing Hospital of China Medical University between May 2016 and March 2018. The trial enrolled 40 women with preeclampsia and 32 women as control without unhealthy living habits or previous abnormal gestation and birth history. All the selected subjects are Chinese Han population. Patients with a preexisting medical disorders history such as congenital hereditary diseases, reproductive organ malformations, malignant tumors, diabetes mellitus, collagen vascular disease, chronic hypertension, renal disease, liver disease, heart disease, any evidence of intrapartum infection as well as any cardiovascular, thyroid, or other endocrinologic disorders were excluded from the study.

### Grouping

In order to analyze the different expression level of GAS5, clinical and laboratory features between preeclampsia and normal gestation as well as early and late-onset preeclampsia, we grouped the subjects as following:

(1) PE (preeclampsia), including two subgroups: (1) ePE (early-onset preeclampsia), this group consisted of women diagnosed with preeclampsia and terminated gestation before 34 weeks (*n* = 20, gestational age: 28 weeks-33 weeks + 6 days); (2) lPE (late-onset preeclampsia), this group consisted of women diagnosed with preeclampsia and terminated gestation after 34 weeks (*n* = 20, 34 weeks -39 weeks).
(2) CON (controls), including two subgroups: (1) eCON (early controls), we used respective gestational age matched pregnant females contrasted with the early-onset preeclampsia patients in order to eliminate the confounded influence of different gestational weeks on the results. This group consisted of women terminated gestation early for placenta previa with placenta accreta spectrum disorders (PASD) accompanied by normal blood pressure during pregnancy (28–36 weeks, *n* = 12) (Sentilhes et al., 2018); (2) lCON (late controls): which consisted of women who had normal pregnancy and did not have any pregnancy complications (34–39 weeks, *n* = 20).

In each case, we confirmed gestational age by the last menstrual period or, if necessary, by ultrasound examination before 14 gestational weeks. The diagnosis criteria of preeclampsia was according to the American College of Obstetricians and Gynecologists and Task Force on Hypertension in Pregnancy (2013), which is defined as hypertension combined with proteinuria or in the absence of proteinuria, new-onset hypertension with the new-onset of any of the following: renal insufficiency, hepatic dysfunction, thrombocytopenia, pulmonary edema and visual or cerebral symptoms. All the participants were delivered by cesarean section to eliminate the interference of different delivery modes on placental samples.

Upon admission, maternal blood pressure data [The measurement method was described previously (American College of Obstetricians and Gynecologists and Task Force on Hypertension in Pregnancy, 2013)] was collected, medical history was recorded and consent was informed. Peripheral blood samples and random midstream urine samples were taken from the gravidas within 24 h after admission to assess the laboratory outcomes. Fasting is required prior to blood being drawn. All the specimens were tested at the clinical laboratory of Shengjing Hospital. Umbilical artery flow S/D value was the ratio of the peak systolic velocity and end-diastolic velocity, which was measured by Doppler ultrasounds following the same data acquisition principle, not later than 24 h after admission. All the participants were followed-up till their discharge and various data were recorded. Adverse fetal outcomes included fetal growth restriction (FGR) (Easter et al., 2017), perinatal asphyxia (Silveira and Procianoy, 2015), stillbirth (Blencowe et al., 2016), and so on. Adverse maternal outcomes were defined as placenta abruption (Elsasser et al., 2010), disseminated intravascular coagulation (DIC) (Wada et al., 2013), eclampsia (American College of Obstetricians and Gynecologists and Task Force on Hypertension in Pregnancy, 2013), hemolysis, elevated liver enzymes, and low platelets (HELLP) syndrome (American College of Obstetricians and Gynecologists and Task Force on Hypertension in Pregnancy, 2013), and so on. All the diagnostic standards according to international criteria were described in the article listed in the References section.

### Placental Tissue Collections

Placental specimens (about 200 mg) were collected within 5 min of placenta delivery during cesarean section. The placental sample was obtained about 2–3 cm in diameter centered around the umbilical cord attachment from maternal surface to fetal surface, which should be avoided calcification and bleeding sites under the aseptic conditions. For the isolation of RNA and protein, only chorionic tissue was collected from the placental central part excluded decidua and amniotic membranes under the observation of morphology. After washing with sterile PBS (phosphate buffer solution) briefly, samples were frozen immediately in nitrogen canister during the operation and stored at −80°C refrigerator in long term.

### Cell Lines and Cell Culture

In this study, HTR-8/SVneo and JEG-3 cell lines were selected. HTR-8/SVneo cell line, human extravillous trophoblast cells, was obtained from Department of Anatomy & Cell Biology, Queen’s University at Kingston, Canada. This cell line owns the characteristics of extravillous cytotrophoblast cells (EVTs) generating from first trimester chorionic villi and is extensively used in the research of abnormal placentation (Huang et al., 2019). JEG-3 cell line, Homo sapiens choriocarcinoma cells, was purchased from the Institute of Biochemistry and Cell Biology, Chinese Academy of Sciences (Shanghai, China). This cell line is similar with EVTs phenotypically and widely applied for the study of cellular invasion and migration (Huang et al., 2019). The HTR-8/SVneo cells were cultured in RPMI 1640 while the JEG-3 cells were in DMEM/F12, supplemented with 10% fetal bovine serum (Biological Industries, Shanghai, China) respectively. The cells were cultured in CO_2_ incubators at 37°C with at 5% CO_2_ in humid environment.

### RNA Isolation and qRT-PCR

Total cellular RNA was extracted from tissues and cell lines using TRIzol reagent (Takara, Dalian, China) according to the manufacturer’s protocol. The concentration and purity of the RNA samples were assayed by Nanodrop (N50 Touch, Implen, Germany). LncRNA was reversely transcribed into complementary DNA (cDNA) using PrimeScript^TM^ RT Master Mix (Takara) according to the manufacturer’s instructions. MicroRNA and mRNA was reversely transcribed into cDNA using the M-MLV reverse transcriptase (Promega, Madison, WI, United States). The cDNA was store at −20°C until use. Quantitative real-time polymerase chain reaction (qRT-PCR) was performed to determine the expression level using a SYBR^®^ Premix Ex Taq^TM^ (Takara) in an ABI 7500 fast fluorescence qPCR amplifier (Applied Biosystems, Foster City, CA, United States) according to the protocols of manufacturer. 18S was the internal reference for GAS5 while U6 was the internal reference for miR-21. Glyceraldehyde 3-phosphate dehydrogenase (GAPDH) was the internal reference for other remaining genes. The sequences of primers were shown in Supplementary Table S1. The 2^–ΔΔ*Ct*^ method were used to determine the relative expression of target as fold changes. The formula applied was as follows: ΔΔCt (Delta–delta C_*t*_ values) = ΔCt _experiment group_ - ΔCt _control group_ and ΔCt = Ct _target gene_ - Ct _internal reference_ (Zhang et al., 2013). All experiments were duplicated three times at least.

### Construction of GAS5 and MiR-21 Expression Vectors

To evaluate the biological function of GAS5 in trophoblast and the correlation between GAS5 and miR-21, we overexpressed and inhibited their expression level using lentivirus vectors (all constructed by Genechem, Shanghai, China). The sequence of GAS5 was synthesized according to the GAS5 full-length sequence (GenBank: NR_002578) and cloned into the lentiviral vector for overexpression. The empty vector was set as control. We also designed and constructed three sets of shRNA for knocking down the GAS5 expression. The target sequences were as follows: (1) 5′-CTTGCCTGGACCAGCTTAA-3′, (2) 5′-GCTCTGGATAGCACCTTAT-3′ and (3) 5′-GGACC AGCTTAATGGTTCT-3′. The sequence of the control vector was 5′-TTCTCCGAACGTGTCACGT-3′. The DNA containing miR-21 (GENE_ID:406991) sequence was amplified by polymerase chain reaction as the template and cloned into the lentiviral vector for overexpression. The empty vector was set as control. Using the mature reverse complementary sequences, miR-21 inhibited oligonucleotides were synthesized. The sequences were 5′-TCAACATCAGTCTGATAAGCTA-3′ and the control sequences were 5′-TTCTCCGAACGTGTCACGT-3′.

### Lentivirus Transfection and Co-transfection

Cell lines were seeded in a 12-well tissue culture plate. After the cell confluency reached about 30 to 50%, cells were transfected with lentivirus vector (MOI = 20) using Eni.S added with 5 μg/mL polybrene transfection reagents (Genechem, Shanghai, China) according to the manufacturer’s instructions. Green fluorescent protein (GFP) expression per well was observed 72 h after lentivirus infection to determine the transfection efficiency. The proportion of green fluorescence represented the transfection efficiency. The stable transfected cells were screened by Puromycin at a dose of 3 μg/mL. Further experiments were carried out when transfection efficiency of cells found to be over 70%.

The two cell lines transfected with GAS5 overexpression lentiviral vector were set as OE (GAS5) and transfected with empty vector were set as NCOE (GAS5). KD (GAS5) was the knockdown group and divided into KD_1_ (GAS5), KD_2_ (GAS5), and KD_3_ (GAS5) according to the three different shRNA sequences. NCKD (GAS5) was the negative control group. The blank control was also set. Under the same grouping principle, the two cell lines transfected with miR-21 overexpression and knockdown lentivirus vectors were divided into OE (miR-21) and KD (miR-21), while NCOE (miR-21) and NCKD (miR-21) were the controls respectively.

The overexpression or the shRNA inhibition experiment can be extended further using “rescue” experiments to verify the correlation between GAS5 and miR-21. Co-transfection method was used as rescue experiment (Roth et al., 2012). GAS5 and miR-21 lentivirus mixed infected the two cell lines respectively. Two different lentivirus were mixed with the same MOI value (MOI = 20) beforehand and transfected cells under the same condition as the transfection protocol mentioned above.

### Cell Proliferation Assay

To measure cell proliferation, we employed MTT assay according to the instructions of manufacturer (Dingguo, Beijing, China). Cells (2,000/well) at the logarithmic growth phase were seeded into 96-well plates and cultured in CO_2_ incubator for 24 h. After adding 20 μL of 5 mg/mL MTT solution to each well, the cells were further incubated for 4 h at 37°C. Following incubation with MTT solution, the supernatant was carefully discarded, and 100 μL of DMSO was added to each well to dissolve formazan crystals. Results were confirmed by Automatic microplate reader (BioTek, Winooski, VT, United States) at 490 nm by measuring the optical density (OD) of each well against a reference blank control. After continuous monitoring for 5 days, the measured days were taken as the x-coordinate and the absorbance as the y-coordinate to plot the cell growth curve reflected the proliferation ability of cells.

### Cell Apoptosis Assays

Apoptosis was determined using the Annexin V-APC apoptosis detection kit (eBioscience, San Diego, CA, United States) according to the manufacturer’s protocol. Twenty four hours post-transfection, cells were suspended and centrifuged for 5 min. The cell pellets were washed (2×) with cold PBS. After washing, 500 μL Annexin V Binding Buffer was added to re-suspended cell pellets. A total of 10 μL Annexin V-APC was added to each sample. After careful mixing of Annexin V-APC, the samples were incubated for 10–15 min at room temperature (25°C) in the dark. Cells were analyzed on a FACS Aria II Flow Cytometer (BD Biosciences, Franklin Lakes, NJ, United States) and analyzed with guava InCyte software (Millipore, Billerica, MA, United States).

### Cell Migration Assay (Scratch Test)

After transfected for 48 h, cells (5 × 10^4^/well) were seeded in 96-well plates. Upon reaching the appropriate confluence (about 90%), monolayers of cells were scratched using a sterile scratch tester. PBS was used to rinse and eliminate the floating cells. Images were captured at different time points (0, 4, 8, 24, and 48 h) under a microscope to assess the migration distance of cells. The cell mobility was described as the ratio between the migration distance at different observation time and the primary width of scratch.

### Cell Invasion Assay

The Transwell chambers with 8.0-μm pores were obtained from Corning (Corning, NY, United States). The Transwell membrane was precoated with 50 μL of Matrigel (1:8 mixed with 4°C medium; Corning) and incubated for 2 h at 37°C incubator to form the permeable filter. The transfected cells were collected and resuspended in 100 μL serum-free medium and then transferred to the upper chambers (2 × 10^4^ cells per well). A medium of 500 μL supplemented with 10% FBS was added to the lower chamber. After incubation for 24 h, the Transwell membrane was fixed with methanol, and stained with crystal violet. Then the number of penetrated cells were counted under a light microscope. Ten fields were selected randomly for counting the transmembrane cells. This assay was used to evaluate the invasion or infiltration capability of cells. The invasion capacity was measured by counting the number of cells entering the lower chamber.

### Western Blot Analysis

The transfected cells were collected and lysed on ice for 20 min using protein lysis buffer RIPA (Beyotime). The samples were centrifuged to collect the supernatant. Following measurement of protein concentration with BCA kit, protein samples were denatured by mixing equal amount of 2 × loading buffer at 100°C for 5 min. An equal amount of protein (30 μg) was loaded in each sample well. The protein samples were separated using SDS-PAGE and transferred to PVDF membranes (Millipore) as described previously (Maryam et al., 2017). The membranes were incubated with diluted primary antibodies (mouse anti-human monoclonal antibody) AKT1 (1:300, ab124341, Abcam, Inc., Cambridge, MA, United States), PI3K (1:100, ab28356, Abcam), PTEN [1:200, #9188, Cell Signaling Technology (CST), Inc., Beverly, MA, United States] MMP9 (1:100, #13667S, CST), TP53 (1:100, #2527, CST), GADPH (1:2000, sc-32233, Santa Cruz, CA, United States) at 4°C overnight. After washing with TBST, the membranes were then incubated with horseradish peroxidase (HRP)-labeled goat anti-rabbit IgG (1:5000, sc-2004, Santa Cruz) and anti-mouse IgG (1: 5000, sc-2005, Santa Cruz). The membranes were immersed in electrochemiluminescence (ECL) luminous liquid (Thermo Fisher Scientific, Waltham MA, United States) and the results were observed in a dark room after developing. Using C300 (Azure Biosystems, Dublin, CA, United States), integrated density values were calculated. Gray value of protein bands was analyzed by Image J, v1.8.0, edition software. The gray value of control group was set as 1, and the relative protein expression level was calculated.

### Statistical Analysis

Data are presented as the mean ± standard deviation (SD). SPSS 21.0 (SPSS, Chicago, IL, United States) and GraphPad Prism 6.01 (GraphPad Software, San Diego, CA, United States) were used for statistical analysis. Statistical significance were assessed by Student’s *t*-test for independent two groups or one-way ANOVA for two groups above, while the Mann–Whitney *U*-test was used for non-parametric independent two-group. Pearson correlation coefficient expressed as ‘r’ was used to evaluate the correlation of two variables. *P* < 0.05 was considered as a statistically significant difference.

## Results

### Clinical and Laboratory Characteristics of Study Population

The characteristics of the Study Population are outlined in Table 1. In maternal age and parity, there were no significant differences between early and late-onset preeclampsia groups and the control group matched (*P* > 0.05) while the gravidity in eCON increased. There was a significant elevation in blood pressure in PE group compared to CON (*P* < 0.05).

**TABLE 1:** Expression level of lncRNA GAS5, Clinical features and Laboratory parameters of the subjects.^a^

	ePE	eCON	lPE	lCON
	(*n* = 20)	(*n* = 12)	(*n* = 20)	(*n* = 20)
GAS5 expression (2^–ΔΔCT^)	1.74 ± 0.52^c^	1.23 ± 0.79	1.29 ± 0.52^d^	1.03 ± 0.24
***Maternal Clinical Features***				
Age (years)	32.1 ± 4.1	30.3 ± 4.9	31.1 ± 5.0	30.3 ± 4.2
Gravidity^f^	2 (1∼4)	3 (1∼6)	2 (1∼4)	2 (1∼3)
Parity^f^	0 (0∼1)	1 (0∼3)	0 (0∼1)	0 (0∼1)
Gestational age at delivery (weeks)	31.6 ± 1.9^c^	33.3 ± 1.8	37.0 ± 1.6^d^	38.2 ± 1.0
Expected treatment time (days)	4.7 ± 3.8^d^	3.1 ± 2.6	2.4 ± 2.1	1.9 ± 1.6
Systolic blood pressure (mmHg)	150.5 ± 25.3^c^	112.7 ± 11.3	153.5 ± 15.4^d^	118.0 ± 6.7
Diastolic blood pressure (mmHg)	95.6 ± 17.4^c^	66.3 ± 6.0	103.4 ± 8.9^d^	74.2 ± 6.5
***Maternal Laboratory Parameters***				
**Parameters described the liver function**:				
Serum Total Protein (g/L)	53.5 ± 6.0^c^	62.9 ± 8.4	59.3 ± 7.9^d^	64.1 ± 4.2
Albumin (g/L)	29.4 ± 3.8^c^	33.9 ± 5.0	32.8 ± 4.9^d^	36.9 ± 2.6
Albumin/Globulin	1.23 ± 0.17^d^	1.18 ± 0.11	1.23 ± 0.17^d^	1.36 ± 0.13
ALT (U/L)	19.8 ± 11.0^c^	9.4 ± 5.5	15.2 ± 8.0	12.4 ± 5.6
AST (U/L)	23.4 ± 8.4^c^	13.6 ± 5.6	22.3 ± 9.9^d^	15.7 ± 5.0
**Parameters described the renal function**:				
Blood urea nitrogen, (mmol/L)	5.8 ± 2.1^c^	3.1 ± 0.9	4.4 ± 1.1^d^	2.9 ± 0.7
Creatinine (μmol/L)	60.9 ± 15.7^d^	52.7 ± 9.5	55.7 ± 10.7^d^	47.3 ± 8.1
Urine acidity (pH value)	6.46 ± 0.59	6.62 ± 0.77	6.31 ± 0.73^d^	6.75 ± 0.62
Proteinuria^e^	3 (1∼4)	1 (0∼4)	2 (0∼4)	0 (0∼1)
Urine specific gravity	1.027 ± 0.01	1.025 ± 0.01	1.024 ± 0.01	1.023 ± 0.01
**Parameters described the coagulation function**:				
Platelet (×10^9^/L)	148.4 ± 67.5^c^	222.8 ± 74.3	182.8 ± 62.0	205.0 ± 54.7
Hemoglobin (g/L)	121.3 ± 23.4^b^	106.2 ± 11.8	131.0 ± 18.4^d^	116.5 ± 11.6
Hematokrit (%)	36.7 ± 7.3	31.9 ± 4.1	39.7 ± 5.9^d^	35.6 ± 3.4
TT, Thrombin time (s)	15.7 ± 0.7^c^	14.7 ± 0.5	15.3 ± 0.6^d^	14.5 ± 0.3
PT, Prothrombin time (s)	9.6 ± 0.7^c^	10.4 ± 0.5	9.8 ± 0.7^d^	10.2 ± 0.5
APTT (s)	26.1 ± 2.8	26.0 ± 1.5	26.9 ± 2.3	26.9 ± 1.9
Fibrinogen (g/L)	3.7 ± 1.0^d^	4.3 ± 1.2	4.5 ± 0.6	4.6 ± 0.7
D-dimer (ng/mL)	837.9 ± 992.4	931.7 ± 737.8	749.7 ± 397.7	806.5 ± 792.5
**Parameters described the thyroid function:**				
fT3, free triiodothyronine (pmol/L)	4.0 ± 0.5^d^	4.0 ± 0.3	4.1 ± 0.5	4.4 ± 0.6
fT4, free thyroxine (pmol/L)	9.6 ± 1.4^d^	10.3 ± 1.4	10.1 ± 1.5^d^	11.1 ± 1.1
TSH (mIU/L)	3.4 ± 2.3^c^	1.5 ± 0.9	2.7 ± 1.3^d^	1.8 ± 0.7
***Fetal Sonographic Evaluation andClinical Features***				
umbilical arteryflow S/D	3.0 ± 0.8^c^	4.3 ± 1.1	2.4 ± 0.4	2.4 ± 0.6
Apgar score^f^ (1min)	7 (0∼10)	7 (3∼10)	10 (7∼10)	10 (9∼10)
Apgar score^f^ (5min)	9 (7∼10)	9 (7∼10)	10 (9∼10)	10 (10∼10)
Birth weight (g)	1433.5 ± 463.9^c^	2107.9 ± 370.7	2810.5 ± 781.0^d^	3277.8 ± 420.0

*ALT, alanine aminotransferase; AST, aspartate aminotransferase; APTT, activated partial thromboplastin time; TSH, thyroid stimulating hormone; S/D, Systolic/Diastolic. ^*a*^Values are shown as mean ± SD. ^*b*^*P* < 0.05, means compared with eCON. ^*c*^*P* < 0.05, means compared with eCON and lCON, respectively. ^*d*^*P* < 0.05, means compared with lCON. ^*e*^Dipsticks method, one “ + ” counts as 1. Non-parametric test Mann–Whitney *U*-test was used. ^*f*^Non-parametric test Mann–Whitney *U*-test was used.*

Various differences can be distinguished between PE and CON:

(1) Elevated or prolonged parameters: AST, ALT, blood urea nitrogen, random midstream urine proteinuria, Thrombin Time (TT), TSH; Declined or shortened parameters: Serum total protein, albumin, platelet, Prothrombin Time (PT), birth weight of neonates. Theses parameters were significantly different among ePE and eCON, ePE, and lCON groups respectively (*P* < 0.05), which might demonstrate the feature of early-onset preeclampsia. The PT evaluates the hemostasis capability and may be shortened in patients with hypercoagulable state. TT extension often occurs in individuals’ pathological anticoagulation state with lower level of fibrinogens (Thornton and Douglas, 2010). The shortened PT and prolonged TT reflected the hypercoagulable state of ePE and the exhaustion of fibrinogen.
(2) Serum Albumin/Globulin ratio, Creatinine, fT4 varied significantly between PE (ePE and lPE) and lCON (*P* < 0.05), indicating the feature of preeclampsia and normal pregnancy.
(3) Fibrinogen, fT3, Apgar score differed significantly between ePE and lCON (*P* < 0.05), but not between lPE and lCON, which might indicate the character of early-onset, meanwhile, could not be excluded the influence of gestational ages.
(4) Urine acidity and Hematokrit were significantly different between lPE and lCON (*P* < 0.05) without differences between ePE and CON (both eCON and lCON) which might be involved in the different pathogenesis of late-onset preeclampsia.

In PE groups (ePE and lPE), there were seven cases (17.5%) of FGR and one case (2.5%) of stillbirth, while placenta abruption occurred in four cases (10%), HELLP syndrome occurred in two cases (5%), Retinal detachment occurred in two cases (5%) and eclampsia was observed in one case (2.5%).

### Upregulation of GAS5 in Preeclamptic Placenta and the Relevance to Clinical Parameters

**FIGURE 1:**
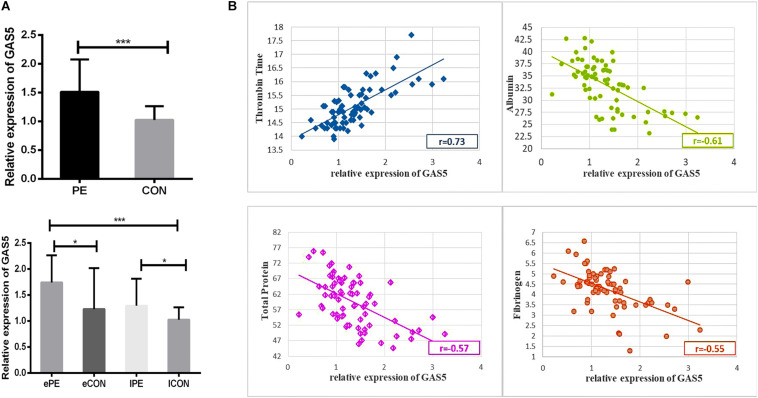
**(A)** The histogram showed the expression of GAS5 elevated in PE groups. The expression level of GAS5 was significantly higher in ePE compared to lCON and eCON. Respectively, the level of GAS5 was also significantly higher in lPE compared to lCON. **(B)** Scatter diagram showing correlation between GAS5 expression level and clinical parameters including TT, Albumin, Serum total protein and Fibrinogen. *P < 0.05, **P < 0.01, ***P < 0.001.

We examined a total of 72 placentas for the expression of GAS5 using qRT-PCR. As shown in Figure 1A and Table 1, expression of GAS5 elevated in PE groups. The expression of GAS5 was significantly elevated in ePE compared to lCON (*P* < 0.001) and eCON (*P* < 0.05), respectively. The level of GAS5 also elevated significantly in lPE compared to lCON (*P* < 0.05).

As shown in Supplementary Table S2, the Pearson’s correlation test demonstrated a significantly positive correlation between expression level of GAS5 and TT (*r* = 0.73, *P* < 0.001). Scatter diagram demonstrated a linear correlation between expression level of GAS5 and TT. A statistically significant and direct negative correlation was also found between GAS5 expression level and Albumin (*r* = −0.61, *P* < 0.001), Serum total protein (*r* = −0.57, *P* < 0.001), Fibrinogen (*r* = −0.55, *P* < 0.001) (Figure 1B).

### The Transfection Efficiency of Vectors in Trophoblast Cells

**FIGURE 2:**
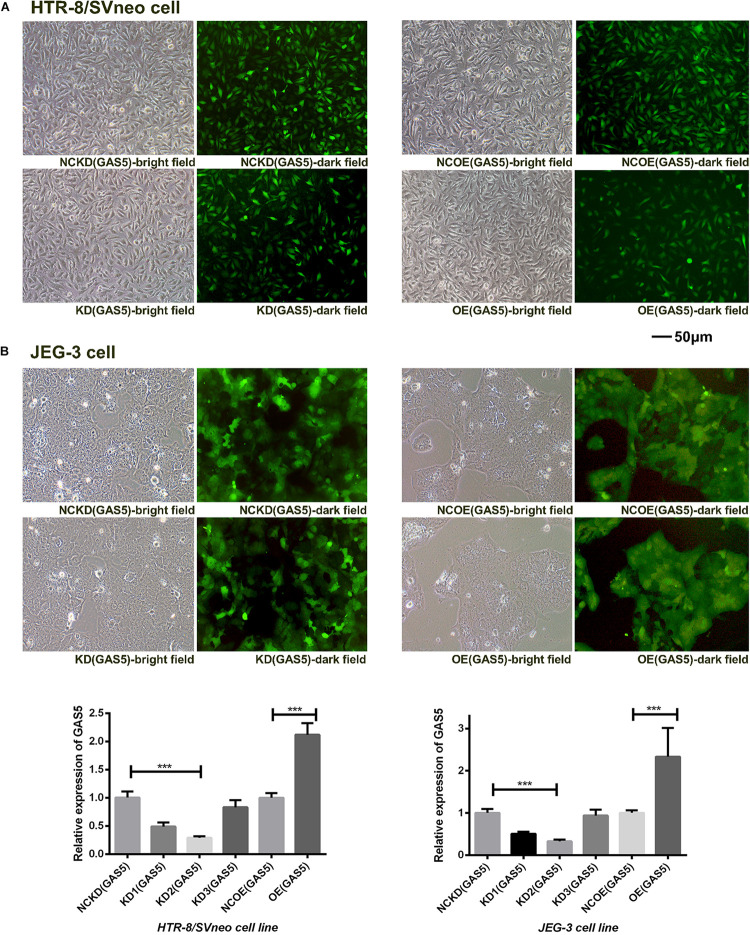
**(A)** The green fluorescence in the dark field indicated the transfection efficiency of the two trophoblast cell lines. **(B)** The expression levels of GAS5 altered by lentiviral vectors in two cell lines. KD_2_ (GAS5) was selected for subsequent experiments as KD (GAS5) with the highest transfection efficiency. *P < 0.05, **P < 0.01, ***P < 0.001.

Both the HTR-8/SVneo and JEG-3 cell lines expressed GAS5 which was detected by qRT-PCR. As shown in Figure 2A, the transfection efficiency was sufficient for the sequent experiment. The three sets of shRNA-GAS5 decreased the expression of GAS5 while the GAS5 overexpression vectors increased GAS5 expression. The two cell lines were noted to exhibit the same trends. In HTR-8/SVneo cell line, GAS5 knocking down efficiency in KD_2_ (GAS5) was 71.4% (*P* < 0.001). In contrast to the control group NCOE (GAS5), the OE (GAS5) was increased by 2.12 times (*P* < 0.001). In JEG-3 cell line, GAS5 Knocking down efficiency in KD_2_ (GAS5) was 67.6% (*P* < 0.001). The OE (GAS5) group was increased by 2.33 times (*P* < 0.05). KD_2_ (GAS5) was selected for subsequent experiments as KD (GAS5). The results above proved that the knockdown and overexpression lentivirus vectors of GAS5 were successfully constructed (Figure 2B).

### GAS5 Influences Proliferation of Trophoblasts but Not Involves in Apoptosis

**FIGURE 3:**
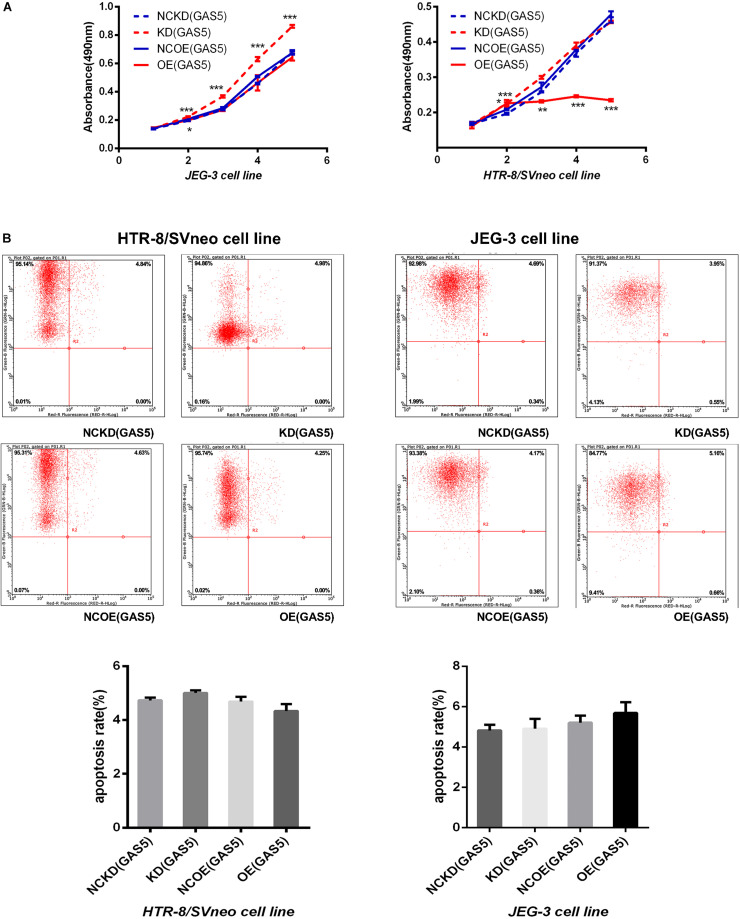
**(A)** The MTT assay indicated that knockdown of GAS5 promoted the proliferation of trophoblast while overexpression of GAS5 inhibited the proliferative ability on the contrary. **(B)** With no significant incidence of apoptosis, GAS5 didn’t influence the apoptosis rate in both of the two cell lines. *P < 0.05, **P < 0.01, ***P < 0.001.

The results (shown in Supplementary Table S3) indicated that the proliferation ability of JEG-3 cells in KD(GAS5) continuously increased compared with the NCKD(GAS5) (*P* < 0.001) while the proliferation ability of HTR-8/SVneo in KD(GAS5) increased on day 2 (*P* < 0.001). On the contrary, OE (GAS5) inhibited the proliferation of HTR-8/SVneo persistently compared with control (*P* < 0.001) while the proliferation of JEG-3 is decreased on day 2 in OE (GAS5) (*P* < 0.05) (shown in Figure 3A).

The apoptosis rate was detected by flow cytometry after overexpression and knockdown of GAS5 in the two cell lines. The results showed that no statistical differences in apoptosis rate occurred in both of the two cell lines (the maximum apoptosis rate in each group was 6.16) (shown in Figure 3B).

### Overexpression of GAS5 Inhibits Trophoblast Migration and Invasion

**FIGURE 4:**
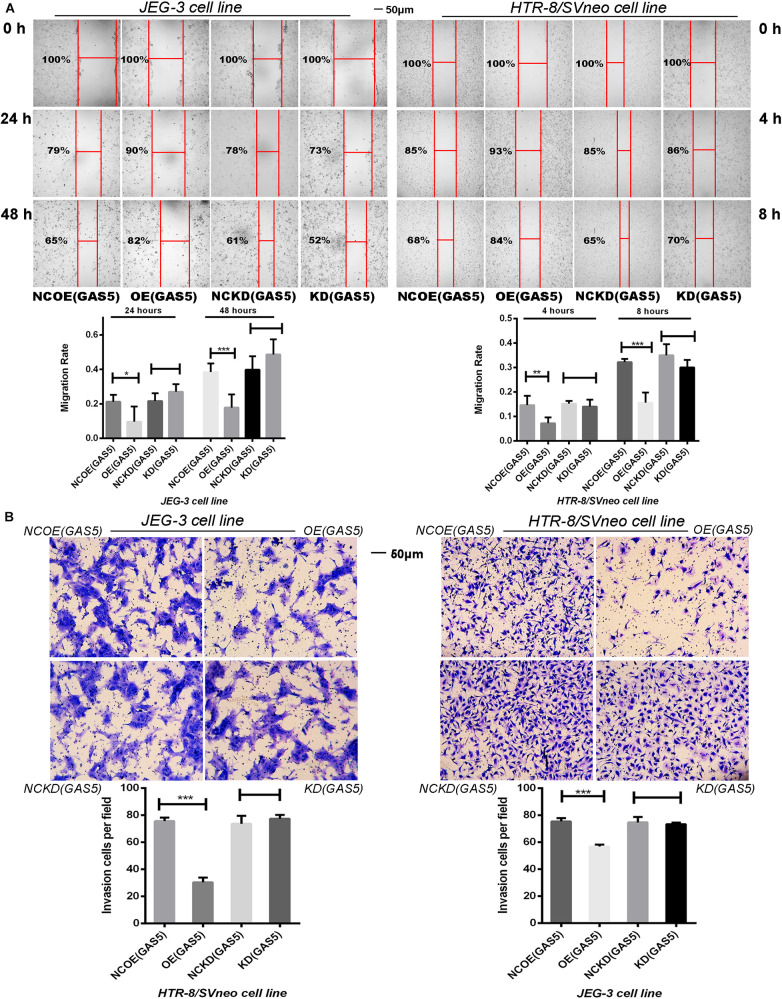
**(A)** The Scratch test demonstrated the migration ability of two cell lines. Overexpression of GAS5 inhibited the migration of trophoblasts while knockdown GAS5 didn’t alter their migration ability. **(B)** Overexpression of GAS5 inhibited the invasion ability of trophoblasts according to transwell assay results, in the meanwhile, knockdown GAS5 didn’t alter their invation ability. *P < 0.05, **P < 0.01, ***P < 0.001.

As depicted in Figure 4A, overexpression of GAS5 significantly reduced the migration ability of JEG-3 cells compared with the NCOE (GAS5). At 24 h post- scratch, the migration rate of OE (GAS5) decreased (*P* < 0.05), and at 48 h post-scratch, the migration rate of the cells was persistently decreased (*P* < 0.001). Meanwhile, after overexpression of GAS5, the migration ability of HTR-8/SVneo cells at 4 and 8 h post-scratch was also inhibited compared with the NCOE (GAS5) (4 h: *P* < 0.01, 8 h: *P* < 0.001). The scratch was healed completely after 24 h. However, no significant difference was observed between KD (GAS5) and NCKD (GAS5) in the two cell lines.

The results of Transwell assay showed that overexpression of GAS5 significantly (*P* < 0.001) reduced the invasion ability of JEG-3 cells, meanwhile, HTR-8/SVneo cell line demonstrated the same trends. The invasion ability of HTR-8/SVneo cells was significantly reduced after overexpression of GAS5 (*P* < 0.001). Nevertheless, no statistic differences demonstrated between KD (GAS5) and NCKD (GAS5) in the two cell lines (Figure 4B).

### Interacting Between GAS5 and MiR-21 in Trophoblast Cells

**FIGURE 5:**
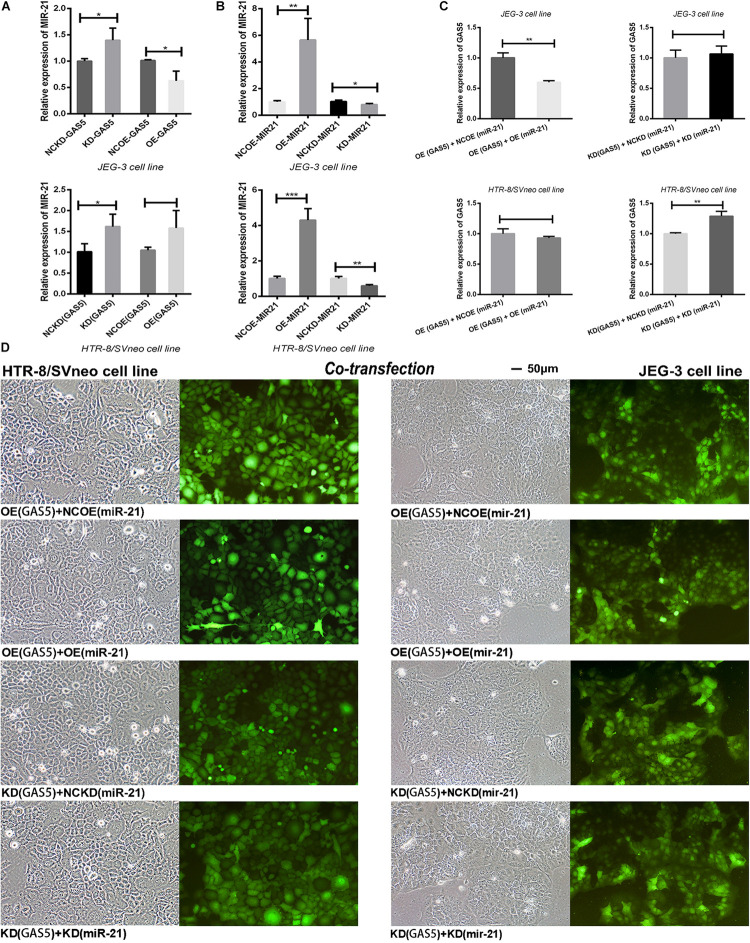
**(A)** After altered the expression of GAS5, the levels of miR-21 demonstrated the reverse tendency. **(B)** The effective transfection rate of miR-21 lentiviral vectors was shown. **(C)** The “rescue” experiment of co-transfection of GAS5 and miR-21 reversed the expression levels of GAS5 in two cell lines. **(D)** The co-transfection efficiency was observed by the green fluorescent in trophoblasts and was adequate for the subsequent experiment. *P < 0.05, **P < 0.01, ***P < 0.001.

After GAS5 lentivirus vectors infected the trophoblast cells, the expression level of miR-21 was firstly detected. The results showed (Figure 5A), in JEG-3 cells, knockdown of GAS5 elevated the expression of miR-21 (*P* < 0.05) compared with the control NCKD (GAS5). Overexpression of GAS5 declined miR-21 expression (*P* < 0.05) compared with the control conversely. In HTR-8/SVneo cells, after knocking down GAS5, the level of miR-21 was also increased (*P* < 0.05). However, the overexpression of GAS5 didn’t alter the level of miR-21 (*P* > 0.05) in HTR-8/SVneo.

Then we detected the transfection efficiency of miR-21 lentivirus vectors by qRT-PCR. The overexpression lentivirus of miR-21 showed high transfection efficiency. In JEG-3, the level of OE (miR-21) was increased about 5.65 times (*P* < 0.01). In HTR-8/SVneo, the expression of OE (miR-21) was increased about 4.30 times (*P* < 0.001). The knockdown lentivirus of miR-21 declined the expression level of miR-21 in KD (miR-21) in both of the HTR-8/SVneo (*P* < 0.01) and JEG-3 (*P* < 0.05) cell lines compared with NCKD (miR-21) controls (Figure 5B).

In order to discern the in-depth interaction between GAS5 and miR-21, we combined GAS5 overexpression lentivirus with miR-21 empty lentiviral vector grouped as OE (GAS5) + NCOE (miR-21). We further combined GAS5 overexpression lentivirus with miR-21 overexpression vector grouped as OE (GAS5) + OE (miR-21) to observe whether the expression of GAS5 could be rescued by miR-21 in two cell lines. With the same principle, KD (GAS5) + NCKD (miR-21) and KD (GAS5) + KD (miR-21) were grouped. The “rescue experiment” showed that after co-transfection of GAS5 and miR-21 expression vectors, compared with OE (GAS5) + NCOE (miR-21), the expression of GAS5 in OE (GAS5) + OE (miR-21) was significantly declined in JEG-3 (*P* < 0.01). In HTR-8/SVneo, the expression level of GAS5 was slightly decreased without statistical significance (*P* > 0.05). Moreover, the expression of GAS5 in KD (GAS5) + KD (miR-21) was statistically elevated compared with KD (GAS5) + NCKD (miR-21) in HTR-8/SVneo (*P* < 0.01). Nevertheless, GAS5 in JEG-3 cell line showed a decrease tendency without statistical difference (*P* > 0.05) (Figures 5C,D).

### Knockdown GAS5 Promotes the Activation of PI3K/AKT Signaling Pathway and the Downstream Proteins

**FIGURE 6:**
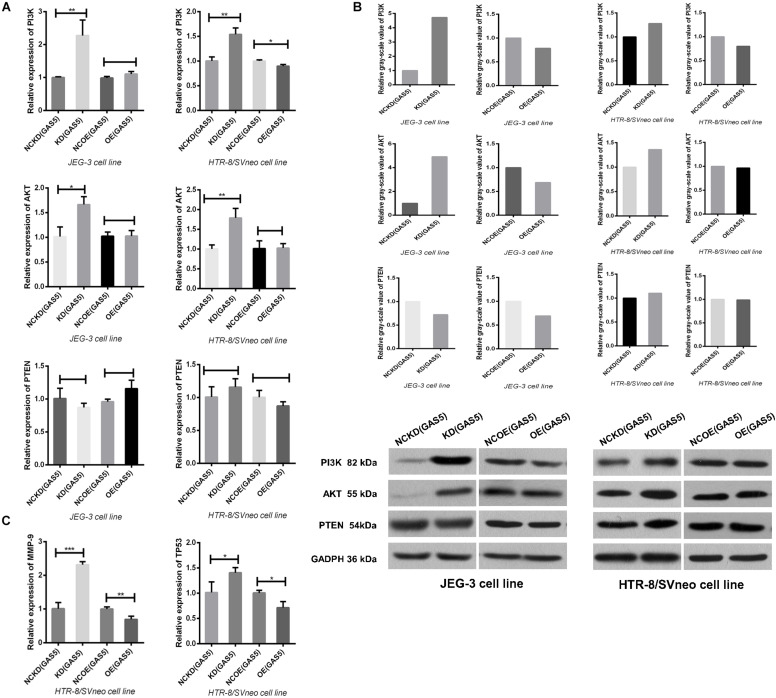
**(A)** Knockdown GAS5 promoted the activation of PI3K/AKT signaling pathway in the two cell lines. In HTR-8/SVneo, overexpression of GAS5 only inhibited the activation of PI3K not involving AKT, while in JEG-3, the PI3K/AKT pathway is not changed. All were analyzed by qRT-PCR assay. **(B)** We also detected the effect of GAS5 on PI3K/AKT pathway by Western Blot. In the two cell lines, knockdown GAS5 promoted the activation and overexpressed GAS5 inhibited this pathway except that the AKT in HTR-8/SVneo was not altered after GAS5 overexpressed, as same as the results detected by qRT-PCR in **(A)**. The PTEN, which was suggested as a target gene of GAS5, was not altered by the two means **(A,B)**. **(C)** The expression of downstream proteins involving MMP9 and TP53 were all elevated after GAS5 knockdown and declined after GAS5 overexpression, which were coincident with the variation of PI3K/AKT signaling pathway in HTR-8/SVneo, verified by qRT-PCR. *P < 0.05, **P < 0.01, ***P < 0.001.

In order to understand the relation between GAS5 and PI3K/AKT signaling pathway further, after altering the expression of GAS5, qRT-PCR and Western Blot (WB) were used to detect the activation of PI3K/AKT signaling pathway, respectively. In JEG-3, qRT-PCR indicated that the expression of PI3K and AKT in KD (GAS5) group increased significantly (*P* < 0.05). In Western blot, consistent with qRT-PCR results, the expression of PI3K and AKT in KD (GAS5) were significantly higher compared to that of NCKD (GAS5) as shown in Figures 6A,B. Under the GAS5 overexpressed condition, the PI3K/AKT is declined that were verified by WB (gray value was 0.78/0.69) without qRT-PCR (*P* > 0.05). In HTR-8/SVneo, with the same alteration tendency, the expression of PI3K/AKT in KD (GAS5) group increased significantly (*P* < 0.01). In Western blot, the expression of PI3K/AKT in KD (GAS5) was 1.28/1.36, the same as qRT-PCR results (Figures 6A,B). When overexpressed GAS5, the PI3K was declined that were verified by both qRT-PCR (*P* < 0.05) and WB (gray value was 0.8) while the AKT expression was not changed by the two means.

Multiple studies have shown that PTEN (phosphatase and tension homology deleted on chromosome 10) has protein and lipid phosphatase activity and can antagonize the activation of PI3K mediated signaling pathway (Pulido, 2018). As a confirmed target gene of miR-21, PTEN is involved in the interaction between GAS5 and miR-21.GAS5 can counteract the reduction of PTEN by miR-21, and play a synergistic role in tumor-suppression with PTEN (Hu et al., 2016; Cao et al., 2017; Tao et al., 2017; Zeng et al., 2019; Zhu et al., 2019). However, the correlation between GAS5 and PTEN in trophoblasts was not observed in this study (Figures 6A,B).

Further, HTR-8/SVneo cell line was selected which was closer to the villous cells in first-trimester placenta. According to the prediction of KEGG (Kyoto Encyclopedia of Genes and Genomes) bioinformatics database, we detected the alteration of the PI3K/AKT signaling pathway downstream proteins MMP9 and TP53, which were closely related with preeclampsia (Gao et al., 2016; Chen and Khalil, 2017), after GAS5 expression changed. The results showed that the expressions of MMP9 and TP53 were all regulated up and down statistically according to the activation of PI3K/AKT signaling pathway by qRT-PCR (Figure 6C). However, due to the low expression level of this two downstream protein in HTR-8/SVneo, no target band was detected in WB.

## Discussion

The causes of preeclampsia remain one of the great medical mysteries of our time (Fisher, 2015). It is evident that enormous progress has been made to understand this complex disorder involving multiple organ systems, using integrative approaches (Palei et al., 2013). According to our current work, GAS5 is a novel lncRNA involving in preeclampsia. The expression of GAS5 was elevated in preeclampsia patients’ placentas and demonstrated a correlation with coagulation parameters-TT and fibrinogen, as well as serum protein level. *In vitro*, the expression of GAS5 is involved in proliferation, migration and invasion, without affecting the apoptosis in trophoblasts. Considering its molecular spongy binding ability, the interaction between GAS5 and miR-21 was firstly and preliminarily detected in trophoblasts. Meanwhile, PI3K/AKT signaling pathway was activated/inactivated by the inhibition/overexpression of GAS5 and the downstream proteins, MMP9 and TP53 were all regulated by GAS5. This is the first report of exploring the role of GAS5 in preeclampsia and expects more subsequent relative researches. Combining with H19, MALAT-1, MEG3 and so on, the involvement of lncRNAs in preeclampsia will become more clear.

Two-stage model hypothesis can better explain the pathogenesis of early-onset preeclampsia. Poor placentation of early-onset in stage 1 was confirmed by morphological and immunohistochemical observation of placental tissue, the violation of trophoblast invasion, and the insufficient remodeling of spiral arteries were expressed. The early-onset type is also called “fetal type” and is more likely linked to fetal growth restriction and stillbirth due to the relevant placental hypoperfusion (Gathiram and Moodley, 2016). Meanwhile, the late-onset is called “maternal type,” which is slightly altered in placental morphology, more linking to preexisting maternal endothelial damage and the cascading widespread vascular spasm, the endpoint of stage 2. The early and late types may share the similar stage 2 process. In this study, the typical laboratory feature of early-onset preeclampsia demonstrated the declined level of serum protein, elevated level of proteinuria, damaging of renal and hepatic function, and hypercoagulation state, as well as low birth weight of the neonates. The results of increasing level of TSH and decreasing level of fT4 in ePE group provided a hint that hypofunction of thyroid might result in early-onset preeclampsia and possibly contribute to the pathogenesis, resembling previous report (Chen et al., 2015).

Pregnancy is a dynamic physiological process, so the gestational age differences may affect the expression level of laboratory outcomes. In this research, we selected the cases with normal blood pressure during pregnancy who terminated pregnancy early due to placenta previa with PASD as the control for early-onset. The deep invasion within the uterine myometrium of PASD is on the contrary of the “shallow placentation” of preeclampsia (Jauniaux et al., 2018). This grouping may eliminate the influence of gestational age and contribute to understand the difference between preeclampsia subtypes. The gravidity in eCON group increased, which was in accordance with the higher risk factors of PASD, for instance of recurrent artificial abortion history, previous cesarean section history and multiple parturition. The interesting result of highest level of umbilical artery flow S/D in eCON was caused by the deep invasion of uterine myometrium increased the circulation amount in intervillous space, leading to increased umbilical arterial post-load and slow diastolic velocity (Jauniaux et al., 2018). The S/D in ePE was higher than lCON without same tendency in lPE compared with lCON, maybe involving in the placental hypoperfusion of early-onset type which was already reported (Adekanmi et al., 2019) and should be compared with the normal pregnancy with the corresponding period for more evidenced proof.

The invasion of trophoblasts and the invasion of tumor cells share similar biological behaviors (West et al., 2018). GAS5 expression which is found to be decreased in tumor tissues (Yu and Hann, 2019) has been proved to increase in preeclampsia placenta in our work firstly, especially with the highest level in early-onset, which is consistent with the pathological feature of insufficient trophoblast invasion. However, placenta development is a sequential process, samples are only available at the end of pregnancy. Researchers are unable to figure out what happened in the initial phase and traced the whole process. Serological samples are easier to access and GAS5 was certificated to be stable in serum (Liang et al., 2016), whether its expression level is parallel to the placenta tissue still need to be proved. So, this elevated expression of GAS5 may reflect the disease state but not fully indicating the relevance to primary pathogenesis. Using the correlation analysis, we found the more prolonged thrombin time (TT), the higher level of GAS5, accompanying with the lower level of fibrinogen. This interesting correlation in our research may reflect the interaction between GAS5 and the pathogenesis of preeclampsia in coagulation dysfunction, while the related mechanisms are still unknown and need further exploration, for instance of the interaction between GAS5 and angiogenesis, the evaluation efficiency of GAS5 on endothelial injury, and so on. Our work revealed the different expression of GAS5 in placentas of preeclampsia patients, simultaneously, possibly offered a clue for the further exploration, on the correlation between GAS5 and endothelial injury cascading coagulopathy. Persistent expanded refinement of the two-stage hypothesis such as “placental syncytiotrophoblast stress” is proposed for the heterogeneous syndrome and unpredictable deterioration (Staff, 2019). Both the hypothesis and the classification are expected to conduct more accurate treatment and better prognosis of the diseases, which still need persistent evaluation.

Normal placental development involves two different types of gestational trophoblasts: extravillous cytotrophoblasts (evCTB or EVT) and villous cytotrophoblasts (vCTB or VT). EVT is responsible for invasion and implantation of endometrium, while VT is crucial for maternal-fetal material exchange and differentiates to secretory syncyotrophblast (STB) (Moser et al., 2018). Clearly, the option of cell lines for the study of preeclampsia *in vitro* is crucial. HTR-8/SVneo cell lines derived from early pregnancy villus explant, which can infinitely proliferate and could be closest to the biological characteristics of villi in early pregnancy, considering as an ideal EVT model simulating the initial phase of the preeclampsia. JEG-3 developed from chorion carcinoma which possess the properties of epithelial cell suggesting it is a good model for studying the migration and invasion properties of EVT. These two cell lines mostly showed the same variation tendency under the regulation of GAS5, there were still some their own unique characters.

Apoptosis induced by GAS5 was certified by several tumor researches (Liu et al., 2018; Gao et al., 2019; He et al., 2019; Zhou et al., 2019), but in this study, we didn’t detect apoptosis inducing effect of GAS5 after transfection. The cell line specificity might be the reason of this discrepancy. The similar result was also found in MCF10A, the immortalized normal breast cell line, without effect of GAS5 on apoptosis basally (Pickard and Williams, 2015), which still needs further verification. Methods to mediate the RNA interference (RNAi) effect covering siRNA (small interfering RNA), shRNA (short hairpin RNA) and bi-functional shRNA. “The shRNA constructs allow for high potency and sustainable effects using low copy numbers resulting in less off-target effects” (Rao et al., 2009). Even the shRNA inhibited GAS5 expression effectively in this experiment, the knockdown of GAS5 couldn’t promote the migration and invasion ability. Maybe the efficiency of interfering vectors was still beyond the reach of GAS5 inhibition threshold to demonstrate visible migration and invasion variation.

MiR-21 is a widely known oncomiR, with the enrichment in human placentas. Inhibited miR-21 reduced the cellular abilities of invasion, migration and proliferation in trophoblast cell lines JEG-3 and HTR-8/SVneo as well as inducing apoptosis in JEG-3 cells (Chaiwangyen et al., 2015). In our experiment, GAS5 and miR-21 were negatively regulated in the two trophoblast cell lines, and co-transfection of GAS5 and miR-21 could rescue the GAS5 expression. The two results, taken together, better illustrated the interaction between GAS5 and miR-21 in trophoblasts. A previous study reported the mechanism of their interaction in breast cancer in detail. After identifying the potential miR-21 binding site in exon 4 of GAS5, the researchers generated a GAS5 mutant clone revealing non-significant inhibition of miR-21 compared with the wild-type, the same as the miR-21 mutant, lost its ability to suppress GAS5. Then, researchers determined the effect of GAS5 on mature miR-21 instead of precursors meaning the post-transcriptional mechanism. Further, researchers performed biotin-labeled GAS5-RNA immunoprecipitation probe using antibody against a key component of the RNA-induced silencing complex (RISC) -AGO2, subsequently identify miR-21 in this GAS5-RISC complex, implying endogenous competitive RNA (ceRNA)-regulatory network, silencing of target mRNAs, including PTEN, TPM1, PDCD4, maspin, RECK, and TIMP3 (Zhang et al., 2013). After this research with very important contributions, lots of subsequent work focused on verifying this theory in different human diseases including our work. Not only miR-21, numerous microRNAs, also covering diverse target genes, are predicted to be interacted with GAS5 according to various databases depending on different algorithms. Considering the microRNA-lncRNA-mRNA changeful expression profile during the pregnancy process, it’s complicated to recognize the interaction between microRNAs, lncRNAs and target mRNAs than imagination. The explanation of the detailed mechanism in our work is insufficient. Relaying on high throughput techniques and validation experiments, further annotation of GAS5 will be obtained gradually, but still a challenging task.

PI3K/AKT signaling pathway is involved in the invasion and EMT (epithelial–mesenchymal transition) process of trophoblast cells in early pregnancy (Xu et al., 2015). It is also involved in the incidence of preeclampsia by regulating VEGF (vascular endothelial growth factor) (Fisher, 2015). In this experiment, GAS5 knockdown activated PI3K/AKT signaling pathway, and increased the expression of downstream protein MMP9 and TP53, the typical molecules involving in cellular migration, invasion, proliferation and apoptosis, perhaps through this route affecting the biological behavior of trophoblast cells. PI3K and AKT usually work closely but there is new evidence that PI3K and AKT can also act independently to build new regulatory networks (Faes and Dormond, 2015). This phenomenon might explain the unparalleled variation between PI3K and AKT after GAS5 overexpression. Changes in downstream protein were only confirmed by qRT-PCR without positive result in Western Blot. It may relate to the lower expression quantity of the proteins, or the synthesis of these downstream require specific time and space which needs appropriate detection time point.

Until now, the pathogenesis of preeclampsia has not been clarified lacking effective predictive index and treatment scheme. With the emergence of RNA sequencing, the number of lncRNAs expressed in human placenta has dramatically expanded, and how lncRNAs involved in preeclampsia have been better understood. The tissue specificity and stability of lncRNAs may provide new biomarkers of the same category for clinical diagnosis. The flexibility and network of expression patterns may also provide a new target for molecular therapy. However, there is still more truth remains to be revealed, more problems to be solved, the off-target effect as a molecular drug and biological toxicity of lncRNA cannot be ignored (Chery, 2016). More and better research tools are needed to assist researchers to understand the dynamic biological feature *in vivo*. In general, the ultimate goal of all efforts is to reduce the incidence and mortality of preeclampsia, and to protect the health of mothers and fetus in long-term.

## Data Availability Statement

The datasets generated for this study are available on request to the corresponding author.

## Ethics Statement

The studies involving human participants were reviewed and approved by the Ethics Committee of Medical Faculty in Shengjing Hospital of China Medical University. The patients/participants provided their written informed consent to participate in this study.

## Author Contributions

CQ and DZ conceived and designed the experiments and analyzed the data. DZ and YH performed the experiments. DZ, CQ, MK, and YB wrote the manuscript. YL and LH collected the placental samples. DZ and FL collected the clinical data.

## Conflict of Interest

The authors declare that the research was conducted in the absence of any commercial or financial relationships that could be construed as a potential conflict of interest.
