# Corrigendum: Long Non-Coding RNA Gas5 is Associated with Preeclampsia and Regulates Biological Behaviors of Trophoblast via MicroRNA-21

**DOI:** 10.3389/fgene.2021.805011

**Published:** 2022-01-03

**Authors:** Dongying Zheng, Yue Hou, Yuanyuan Li, Yue Bian, Muhanmmad Khan, Fan Li, Ling Huang, Chong Qiao

**Affiliations:** ^1^ Department of Obstetrics and Gynecology, Shengjing Hospital, China Medical University, Shenyang, China; ^2^ Department of Obstetrics and Gynecology, Second Affiliated Hospital of Dalian Medical University, Dalian, China; ^3^ Key Laboratory of Maternal-Fetal Medicine of Liaoning Province, Shenyang, China; ^4^ Key Laboratory of Obstetrics and Gynecology of Higher Education of Liaoning Province, Shenyang, China; ^5^ Research Center of China Medical University Birth Cohort, Shenyang, China; ^6^ Department of Zoology, University of the Punjab, Lahore, Pakistan

**Keywords:** long non-coding RNA, Gas5, preeclampsia, placenta, trophoblast, miR-21, early-onset

In the original article, there was a mistake in Figure 4B as published. “The microscopic image of NCKD(GAS5) group was wrongly numbered and overlapped with the microscopic image of NCOE(GAS5) group, the HTR-8/SVneo cell line”. The corrected Figure 4 appears below.

**FIGURE 4:**
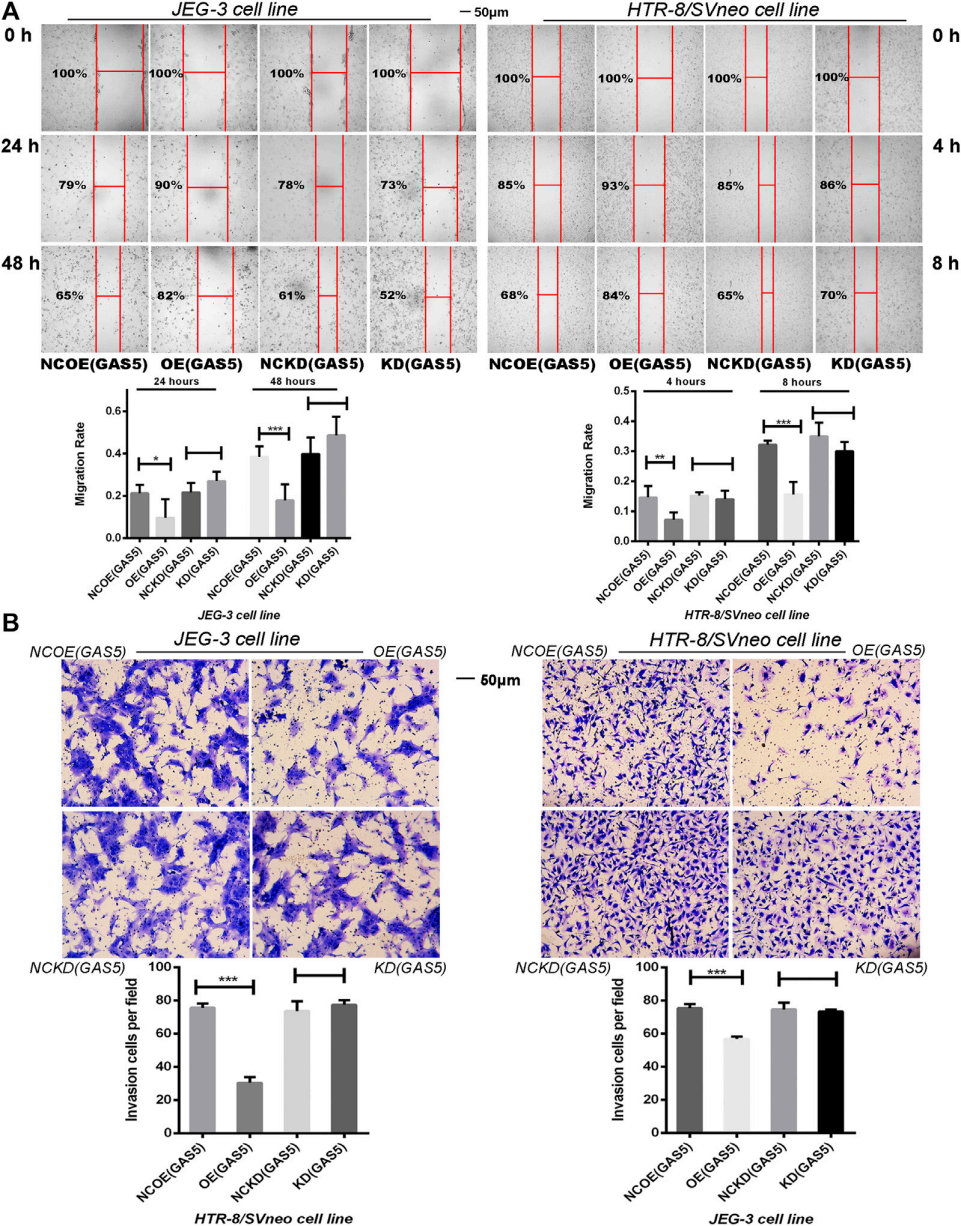
**(A)** The Scratch test demonstrated the migration ability of two cell lines. Overexpression of GAS5 inhibited the migration of trophoblasts while knockdown GAS5 didn’t alter their migration ability. **(B)** Overexpression of GAS5 inhibited the invasion ability of trophoblasts according to transwell assay results, in the meanwhile, knockdown GAS5 didn’t alter their invation ability. *p < 0.05, **p < 0.01, ***p < 0.001.

The authors apologize for this error and state that this does not change the scientific conclusions of the article in any way. The original article has been updated.

