# Supplementary material for: Long Non-coding RNA Gas5 Is Associated With Preeclampsia and Regulates Biological Behaviors of Trophoblast via MicroRNA-21
Source: Front Genet. 2020 Mar 3;11:188. doi: 10.3389/fgene.2020.00188 (PMC7063462; doi:10.3389/fgene.2020.00188)
Supplement: Supplementary file 1 [file Data_Sheet_1.docx]

Supplementary Table S1. Primers used in RT-qPCR.^a^

|  | Primer | Forward (5'‑3') | Reverse (5'‑3') |
| --- | --- | --- | --- |
| Target gene | GAS5 | TGCAGTGTGGCTCTGGATAG | CTAATGCCTGTGTGCCAATG |
| [Reference](javascript:;) [gene](javascript:;) | 18S | ATCCTCAGTGAGTTCTCCCG | CTTTGCCATCACTGCCATTA |
| Target gene | AKT1 | TCTTTGCCGGTATCGTGT | TGTCATCTTGGTCAGGTGGT |
|  | PIK3CB | TATTTGGACTTTGCGACAAGACT | TCGAACGTACTGGTCTGGATAG |
|  | PTEN | AAGACCATAACCCACCACA | ATTACACCAGTTCGTCCCT |
|  | MMP9 | GCACCACCACAACATCAC | ACCACAACTCGTCATCGTC |
|  | TP53 | CCTCCTCAGCATCTTATCC | ACAAACACGCACCTCAAA |
| [Reference](javascript:;) [gene](javascript:;) | GAPDH | TGACTTCAACAGCGACACCCA | CACCCTGTTGCTGTAGCCAAA |

^a^ The primers sequences of miR-21 and U6 were only offered by catalog number(Cat. No.). (Ribobio, Guangzhou, China)

U6: reverse transcription primer Cat. No.: SSD904071008; upstream primer Cat. No.: SSD0904071006; downstream primer Cat. No.: SSD0904071007.

miR-21: reverse transcription primer Cat. No.: SSD809230239; upstream primer Cat. No.: SSD809230931; downstream primer Cat. No.: SSD089261711.

Supplementary Table S2. The expression of GAS5 in placental tissue correlated with clinical parameters ^a^

| indicators | Pearson correlation  (r values) |
| --- | --- |
| TT | 0.73^***^ |
| Albumin | -0.61^***^ |
| Serum total protein | -0.57^***^ |
| Fibrinogen | -0.55^***^ |
| Blood urea nitrogen | 0.48^***^ |
| proteinuria | 0.45^***^ |
| Platelet | -0.44^***^ |
| fT3 | -0.33^**^ |
| Creatinine | 0.29^*^ |
| Albumin/Globulin | -0.28^*^ |
| fT4 | -0.24^*^ |
| PT | -0.23^*^ |

^a^ r≤0.3, the correlation was not significant; 0.3 < r≤0.8, there was a direct correlation; R > 0.8, strongly significant correlation.

^*^P < .05, ^**^ P < .01, ^***^ P < .001

Supplementary Table S3. OD490 Values of MTT Assay

|  | HTR-8/SVneo cell line (n=3) | | | |
| --- | --- | --- | --- | --- |
| time(day) | NCKD(GAS5) | KD(GAS5) | NCOE(GAS5) | OE(GAS5) |
| 1 | 0.168 ± 0.002 | 0.167 ± 0.003 | 0.169 ± 0.002 | 0.161 ± 0.004 |
| 2 | 0.196 ± 0.002 | 0.225 ± 0.002 | 0.209 ± 0.001 | 0.226 ± 0.005 |
| 3 | 0.258 ± 0.001 | 0.382 ± 0.046 | 0.272 ± 0.007 | 0.231 ± 0.002 |
| 4 | 0.368 ± 0.006 | 0.386 ± 0.045 | 0.379 ± 0.003 | 0.246 ± 0.002 |
| 5 | 0.462 ± 0.004 | 0.378 ± 0.046 | 0.478 ± 0.005 | 0.235 ± 0.002 |
|  | JEG-3 cell line (n=3) | | | |
| time(day) | NCKD(GAS5) | KD(GAS5) | NCOE(GAS5) | OE(GAS5) |
| 1 | 0.139 ± 0.002 | 0.141 ± 0.002 | 0.140 ± 0.004 | 0.147 ± 0.001 |
| 2 | 0.196 ± 0.001 | 0.224 ± 0.001 | 0.209 ± 0.001 | 0.198 ± 0.003 |
| 3 | 0.275 ± 0.004 | 0.367 ± 0.004 | 0.285± 0.004 | 0.271 ± 0.004 |
| 4 | 0.458 ± 0.003 | 0.630 ± 0.009 | 0.507 ± 0.003 | 0.463 ± 0.031 |
| 5 | 0.678 ± 0.009 | 0.862 ± 0.006 | 0.676 ± 0.005 | 0.644 ± 0.014 |
